# Macrofilaricidal efficacy of single and repeated oral and subcutaneous doses of flubendazole in *Litomosoides sigmodontis* infected jirds

**DOI:** 10.1371/journal.pntd.0006320

**Published:** 2019-01-16

**Authors:** Marc P. Hübner, Alexandra Ehrens, Marianne Koschel, Bettina Dubben, Franziska Lenz, Stefan J. Frohberger, Sabine Specht, Ludo Quirynen, Sophie Lachau-Durand, Fetene Tekle, Benny Baeten, Marc Engelen, Charles D. Mackenzie, Achim Hoerauf

**Affiliations:** 1 Institute for Medical Microbiology, Immunology and Parasitology, University Hospital of Bonn, Bonn, Germany; 2 Drugs for Neglected Diseases initiative, Geneva, Switzerland; 3 Janssen R&D, Janssen Pharmaceutica, Beerse, Belgium; 4 Neglected Tropical Disease Support Center, Task Force for Global Health, Atlanta, GA, United States of America; 5 German Center for Infection Research (DZIF), partner site Bonn-Cologne, Bonn, Germany; University of Liverpool, UNITED KINGDOM

## Abstract

Flubendazole (FBZ) is highly efficacious against filarial nematodes after parenteral administration and presents a promising macrofilaricidal drug candidate for the elimination of onchocerciasis and other filariae. In the present study the efficacy of a newly developed bioavailable amorphous solid dispersion (ASD) oral formulation of FBZ was investigated in the *Litomosoides sigmodontis* jird model. FBZ was administered to chronically infected, microfilariae-positive jirds by single (40mg/kg), repeated (2, 6 or 15mg/kg for 5 or 10 days) oral (OR) doses or single subcutaneous (SC) injections (2 or 10mg/kg). Jirds treated with 5 SC injections at 10mg/kg served as positive controls, with untreated animals used as negative controls. After OR doses, FBZ is rapidly absorbed and cleared and the exposures increased dose proportionally. SC administered FBZ was slowly released from the injection site and plasma levels remained constant up to necropsy eight weeks after treatment end. Increasing single SC doses caused less than dose-proportional exposures. At necropsy, all animals receiving 1x or 5x 10mg/kg SC FBZ had cleared all adult worms and the 1x 2mg/kg SC treatment had reduced the adult worm burden by 98%. 10x 15mg/kg OR FBZ reduced the adult worm burden by 95%, whereas 1x 40mg/kg and 5x 15mg/kg OR reduced the worm burden by 85 and 84%, respectively. Microfilaremia was completely cleared at necropsy in all animals of the SC treatment regimens, while all oral FBZ treatment regimens reduced the microfilaremia by >90% in a dose and duration dependent manner. In accordance, embryograms from female worms revealed a FBZ dose and duration dependent inhibition of embryogenesis. Histological analysis of the remaining female adult worms showed that FBZ had damaged the body wall, intestine and most prominently the uterus and uterine content. Results of this study demonstrate that single and repeated SC injections and repeated oral administrations of FBZ have an excellent macrofilaricidal effect.

## Author summary

Onchocerciasis and lymphatic filariasis are debilitating human diseases that are caused by filarial nematodes leading to blindness and severe dermatitis (onchocerciasis) or lymphedema (elephantiasis) and hydroceles. Current mass drug administration (MDA) programs are restricted to the use of drugs that target the filarial offspring, the microfilariae, and lead to a temporary sterilization of the female adult worms that requires annual to bi-annual MDA for the life span of the fertile adult worms. With lower endemicity, the cost-effectiveness of community-directed MDA is reduced, requiring alternative treatment strategies to ultimately eliminate these filarial infections. Therefore, new drugs targeting the adult worms are required for achieving elimination of those filarial diseases, to provide options in areas of drug resistance, and as an approach in areas of *Loa loa* co-endemicity, where life-threatening severe adverse events may occur through the use of microfilaricidal drugs. Such new drugs should have a macrofilaricidal effect, i.e. kill the adult worms or lead to a permanent sterilization. Furthermore, these drugs should be preferably administered as an oral formulation or by a single parenteral administration. In the current study we tested a new oral formulation of flubendazole using the rodent filarial nematode *Litomosoides sigmodontis*, and have demonstrated excellent macrofilaricidal efficacy.

## Introduction

Lymphatic filariasis and onchocerciasis are neglected tropical diseases that are caused by parasitic filarial nematodes and present a major public health and economic problem in endemic countries as is demonstrated by 2.78 million and 0.5 million Disability Adjusted Life Years (DALYs), respectively [1]. Lymphatic filariasis, commonly known as elephantiasis, is caused by *Wuchereria bancroftii*, *Brugia malayi*, and *Brugia timori*, and manifestations of the disease include lymphedema and hydrocele leading to disabilities, physical impairment, subsequent income loss and social stigmatization [2]. Onchocerciasis, commonly known as river blindness, is caused by the filarial nematode *Onchocerca volvulus* with immune-inflammatory reactions towards the microfilariae (MF) provoking dermatitis, vision impairment and blindness [3]. WHO is currently targeting the elimination of lymphatic filariasis and onchocerciasis. Past efforts to eliminate both diseases relied on the mass drug administrations (MDA) of ivermectin (IVM) for onchocerciasis and IVM plus albendazole (ALB) for lymphatic filariasis in sub-Saharan Africa and diethylcarbamazine (DEC) plus ALB for lymphatic filariasis outside of Africa [2, 4]. Both DEC and IVM target MF and temporarily sterilize female adult worms [2, 5]. However, due to their lack of macrofilaricidal efficacy or of induction of permanent sterilization, IVM and DEC have to be given on an annual or bi-annual basis for the life span of the adult worms [2]. Recently a triple therapy with IVM, DEC and ALB was explored for the treatment of lymphatic filariasis outside of Africa, which could accelerate the elimination of lymphatic filariasis provided a population coverage >65% were achieved [6], as this new therapy significantly improves MF clearance and maintenance of amicrofilaremia compared to an MDA regimen with DEC and ALB [6, 7]. However, modelling studies suggest that in areas of lower prevalence of lymphatic filariasis the impact on the reduction of MDA treatment rounds will be less prominent [6]; in addition, future studies will need to confirm whether it is possible to safely administer this triple therapy in areas co-endemic for onchocerciasis in Africa, where DEC is currently not used due to the risk of onchocerciasis patients of developing ocular damage [2]. With regard to onchocerciasis, MDA programs, generally using biannual ivermectin, have been successful in almost achieving elimination of this disease in the Americas with less than 1% of the originally infected population still carrying the parasite [8, 9]. The WHO has also refocused onchocerciasis programs from control as public health problem to elimination for endemic countries in Africa, the vast majority of the global burden of this disease [9]. As endemic countries achieve low levels of endemicity the cost-effectiveness of community-directed MDA treatments is reduced and alternative treatment strategies are required to ultimately eliminate these filarial infections. This need for alternate treatment strategies is further supported by the occurrence of foci with suboptimal response to IVM [10] and regions in Africa co-endemic for *Loa loa*, where microfilaricidal drugs such as IVM can induce life-threatening severe adverse events due to rapid killing of *L*. *loa* MF, and test and treat strategies are currently required to safely exclude individuals with high *L*. *loa* microfilaremia [9]. Such alternate treatment strategies include the identification of compounds with a macrofilaricidal or adult-worm sterilizing efficacy that could be used for treatment of the remaining infected patients [11, 12].

Flubendazole (FBZ) is a methylcarbamate benzimidazole that inhibits glucose uptake and microtubule formation and is highly efficacious against human gastrointestinal nematodes [13–15]. In addition, historical data indicate that subcutaneous injections of flubendazole provide a macrofilaricidal effect that clears up to 100% of the adult filariae and is therefore frequently used as a positive control in experimental animal studies of filariasis analyzing direct-acting compounds [16–18]. Efficacy of 5 intramuscular injections of 750mg FBZ was already shown in a historic field trial in 10 onchocerciasis patients to reduce microfilaridermia and to clear intracorneal microfilariae 6 months after treatment [19]. Those historical studies suggest that FBZ has an excellent efficacy after repeated parenteral administration against filarial nematodes. Since the current target product profile agreed upon by expert groups together with the Bill & Melinda Gates Foundation and the Drugs for Neglected Disease initiative (DNDi) favors an oral administration or alternatively a single parenteral administration, Janssen developed the new Janssen Bend 1/9 amorphous solid dispersion (ASD) bioavailable oral formulation of FBZ.

In this study we compared the efficacy of different oral and subcutaneous FBZ regimens in jirds naturally infected with the parasitic filarial nematode *Litomosoides sigmodontis*. *L*. *sigmodontis* was chosen, as it is closely related to human pathogenic filariae and develops patent infections in jirds with microfilariae present in the peripheral blood and adult worms residing in the thoracic cavity for more than one year [20, 21]. Similar to most human-pathogenic filariae, *L*. *sigmodontis* contains endosymbiotic *Wolbachia* bacteria and the *L*. *sigmodontis* mouse model was used to demonstrate the efficacy of *Wolbachia* targeting drugs like doxycycline [22–24], which subsequently led to human studies identifying doxycycline as individual therapy for onchocerciasis and lymphatic filariasis patients [25–27]. Treatment with known microfilaricidal compounds like IVM and DEC clear *L*. *sigmodontis* microfilariae *in vivo* without impacting the adult worm burden, whereas subcutaneous—but not oral—FBZ regimens successfully clear *L*. *sigmodontis* adult worms [28, 29]. Therefore, the *L*. *sigmodontis* rodent model overcomes the lack of a rodent model with human-pathogenic *O*. *volvulus* filariae. However, host and filarial species dependent differences in the susceptibility to micro- and macrofilaricidal compounds do exist, as was shown in several comparable analysis with *L*. *sigmodontis*, *Acanthocheilonema viteae*, *Brugia pahangi* and *Brugia malayi* infected animals [28–30]. Therefore, the present study is part of a FBZ PLOS NTD collection, analyzing the efficacy of OR and SC FBZ regimens in jirds infected with *L*. *sigmodontis* and *B*. *pahangi*, as well as scid mice implanted with *Onchocerca ochengii* in order to obtain a comprehensive overview of the efficacy of OR and SC FBZ regimens in different filarial species to predict, as good as possible, the potential efficacy against human pathogenic filariae.

Results from our study demonstrate that single and repeated subcutaneous injections as well as repeated oral administrations of FBZ have an excellent macrofilaricidal efficacy, clearing >90% of the adult worms. FBZ treatment inhibited the embryogenesis of female adult worms and histopathological analysis suggest arguably irreversible damage of the female uteri and uterine contents.

## Methods

### Ethics statement

All animal experiments were approved by the Landesamt für Natur, Umwelt und Verbraucherschutz, Köln, Germany, (AZ 84–02.04.2015.A507) and conducted in accordance with the European Union Directive 2010/63/EU. Animals were checked for food, water and welfare daily and scored once a week. For scoring, the weight of the animals was determined and their wellbeing analyzed. A score of A-C was assigned regarding the severity of any symptoms considering appearance, weight loss, injuries and behavioral changes. Assignment of A required daily observation of the symptoms, B required consultation of a veterinary or project manager while C required immediate humane euthanization.

### Animals

For the experiment, 8-week old female jirds (*Meriones unguiculatus*) were obtained from Charles River Labs (US) and housed in individually ventilated Typ IV S cages, according to animal welfare guidelines. Autoclaved water and food (V1124-300, ssniff Spezialdiäten GmbH, Soest, Germany) was provided *ad libitum*. Additionally, animals were provided with nestlets and wooden sticks for enriched environment. Light/dark cycle was 12 hour each. The temperature was maintained at 23°C and humidity was maintained below 65%.

### Infection with *L*. *sigmodontis*, group assignment and treatment

The jirds were infected after two weeks of acclimatization by exposure to mites (*Ornithonyssus bacoti*) as vectors to transmit *L*. *sigmodontis* L3 larvae as previously described [31]. For infection, jirds were divided in 2 groups, which were infected two weeks apart with the same batch of mite-containing bedding per infection time point. All treatment groups, except for the positive controls, contained equal numbers of animals from both infection time points.

Six days prior to treatment start (10 and 12 weeks post infection) 10μl of peripheral blood was drawn from the vena saphena and directly transferred into 500μl Hinkelmann solution (0.5% Eosin Y, 0.5% Phenol, 0.185% Formaldehyde in aqua dest). Microfilarial loads were determined by microscopic analysis. Only microfilaremic animals were used in the study and these were allocated to the different treatment groups.

Treatment of the animals was started either 76 or 90 days post infection (dpi) and animals received either oral gavages or subcutaneous injections of FBZ. Oral FBZ (HPMCAS-H, Lot nr. BREC-1113-036) was formulated in a vehicle of 0.5% w/v Methocel A4M and FBZ for subcutaneous application in 0.5% w/v Hydroxyethylcellulose (Sigma 434965) in demineralized water and 0.1% Tween80. The mean weight of jirds at initiation of dosing was 71.7g +/- 5.9g.

A total of 11 experimental groups were included in this study with each group consisting of 11–12 animals (S1 Table). Untreated animals served as negative control. Animal numbers per group were based on power calculations, showing that 12 animals per group will provide a power of 89.6% to detect a reduction in the adult worm burden by 80% with an α-value of 5%. The positive control group received FBZ subcutaneously at 10mg/kg once per day for 5 consecutive days. Experimental groups consisted of 3 single dose groups: one test group received one dose of 40mg/kg FBZ by oral gavage, the other two groups received a single dose of 2 or 10mg/kg FBZ subcutaneously. Additional experimental groups included six repeated dose groups with three groups receiving daily doses for 5 consecutive days and the other three groups daily doses for 10 consecutive days at 2, 6, and 15mg/kg FBZ.

### Pharmacokinetics

Blood samples were taken during the dosing period using a sparse sampling approach with 3–4 animals per time point and group. Sparse sampling for the oral repeated dosing groups included one single time point 2h after dosing at day 1–4 for group 3-4-5 and 11, day 1–9 for group 6-7-8. After last treatment, on day 1 for single dose group, day 5 for 5-day treated groups, day 10 for 10-day treated groups, samples were taken at 0.5h, 1h, 2h, 4h, 8h, and 24h. Sparse sampling for the single dose subcutaneous treated groups were performed at 1h, 3h, 8h, 24h, 48h, 72h, 96h, 120h, week 2, week 3, week 4, week 5, week 6, week 7, week 8. Sparse sampling for the 5-times subcutaneous treated group occurred on day 1, day 2, day 3, day 4 (here 1h, 3h, 8h, 24h), day 7, day 8, day 9, day 10, week 3, week 4, week 5, week 6, week 7, and week 8.

Plasma was obtained by centrifugation at 1900g for 10min and analysis of the plasma samples was done at the PD&S-PDM regulated bioanalysis department (J&J PRD, Beerse, Belgium). Plasma samples were analysed for FBZ (JNJ-161941) and its metabolites JNJ-114699 (hydrolysed FBZ, H-FBZ) and JNJ-1809600 (reduced FBZ, R-FBZ) using a qualified research LC-MS/MS method. The samples were subjected to a selective sample cleanup, followed by HPLC-MS/MS. HPLC separation was done using non-chiral reversed phase liquid chromatography. Subsequent MS/MS analysis was performed using triple quadrupole mass spectrometry in the Multiple Reaction Monitoring (MRM) mode, optimized for the compounds. Samples were quantified against calibration curves and QC samples prepared to cover the concentration range of the study samples. The curves and QC samples were prepared in the same matrix as the study samples. In the repeated dosing groups, the 24h concentrations were used as the 0h concentrations. A non-compartmental analysis using the lin/log trapezoidal rule with lin/log interpolation was used for all data. The following pharmacokinetic parameters were calculated: C_max_, T_max_, and AUC values. Dose-proportionality and time differences were also evaluated.

### Parasite recovery, embryograms and histological assessment of filariae

Jirds were euthanized by an overdose of isoflurane at 8 weeks after treatment when adult worms were collected from the pleural cavity and peritoneum, checked for motility and were counted. Untreated animals were euthanized on the same day as the 5 day treated animals. Female adult worms from each jird were preserved in 10% formalin for 24h and stored in 60% ethanol for subsequent embryogram analysis. If present, 4 randomly selected intact female worms were analyzed for embryogenesis per jird. For embryograms single worms were homogenized in 20μl of Hinkelmann solution and 80μl PBS. The embryonal stages enumerated were eggs, morulae, pretzel and stretched MF as described before [32].

Furthermore, the number of MF isolated from the blood and thoracic cavity at necropsy was analyzed microscopically. Therefore, blood was drawn from the vena saphena and 10μl of the peripheral blood were diluted in 300μl of Hinkelmann solution. For MF counts in the thoracic cavity, the pleura was rinsed with 1ml RPMI medium and 200μl of this lavage has been transferred to 500μl Hinkelmann solution [33].

For histological assessment of the remaining female adult worms, one randomly selected intact individual worm per jird, if present, was selected and worms were processed for sectioning by embedding in HistoGel (Thermo Scientific Richard-Allan Scientific—American MasterTech, Waltham, MA, US), an aqueous gel composition, as coiled entities and sectioned by standard histological section preparation procedures [34–36] they were stained by hematoxylin and eosin for assessment. Two major criteria were assessed: firstly, damage to the three major structures of the worm (i.e. body wall, the intestine, and the reproductive organs and their contents), and secondly the presence of fully developed MF present in the uterus. Damage of the female worms was scored using a 4-point scale with 0 = normal morphology, 1 = minor pathology, 2 = significant damage, 3 = extensive damage, 4 = loss of contents. Damage present in worms is not always uniformly distributed throughout the parasite and so the score allocated to a worm was based on an overall assessment of the status of the sections observed, and the presence of the scored level of change or damage in a significant proportion of the worm. The presence of MF in the uterus was also recorded; the scores used were—when present but in a state of degeneration (= 1) or when morphologically normal (= 2).

All individuals observing the jirds for signs of ill health and all individuals responsible for counting the numbers of adult worms and MF recovered from jirds were blinded as to the treatment. Also, the individuals who assessed parasite vitality and structural integrity were blinded as to treatment group.

### Statistics

To determine statistical significant changes in the number of adult parasites enumerated at necropsy between each dose group (and positive control group) and the negative control group the data was first checked for normality, using the Shapiro-Wilk test, and homoscedasticity with the Levene's test. As the data did not pass both tests, even after logarithmic transformation, Dunn’s test was used on the original data. The positive control group was compared to the control group by means of a one-sided t-test. All tests were performed at a significance level of 5%. Once there was a significance difference between a dose and control groups, then the percent effectiveness was calculated using geometric mean (GM) as follows:

$$\%\;effectiveness\;of\;a\;dose\;group=\frac{100[GM\;of\;no.\;of\;parasites\;in\;control\;group-GM\;of\;no.\;of\;parasites\;in\;dose\;group]\;}{GM\;of\;no.\;of\;parasites\;in\;control\;group}$$

## Results

### Oral and subcutaneous FBZ treatment reduces the adult worm burden in a dose and treatment duration dependent manner

**Fig 1:**
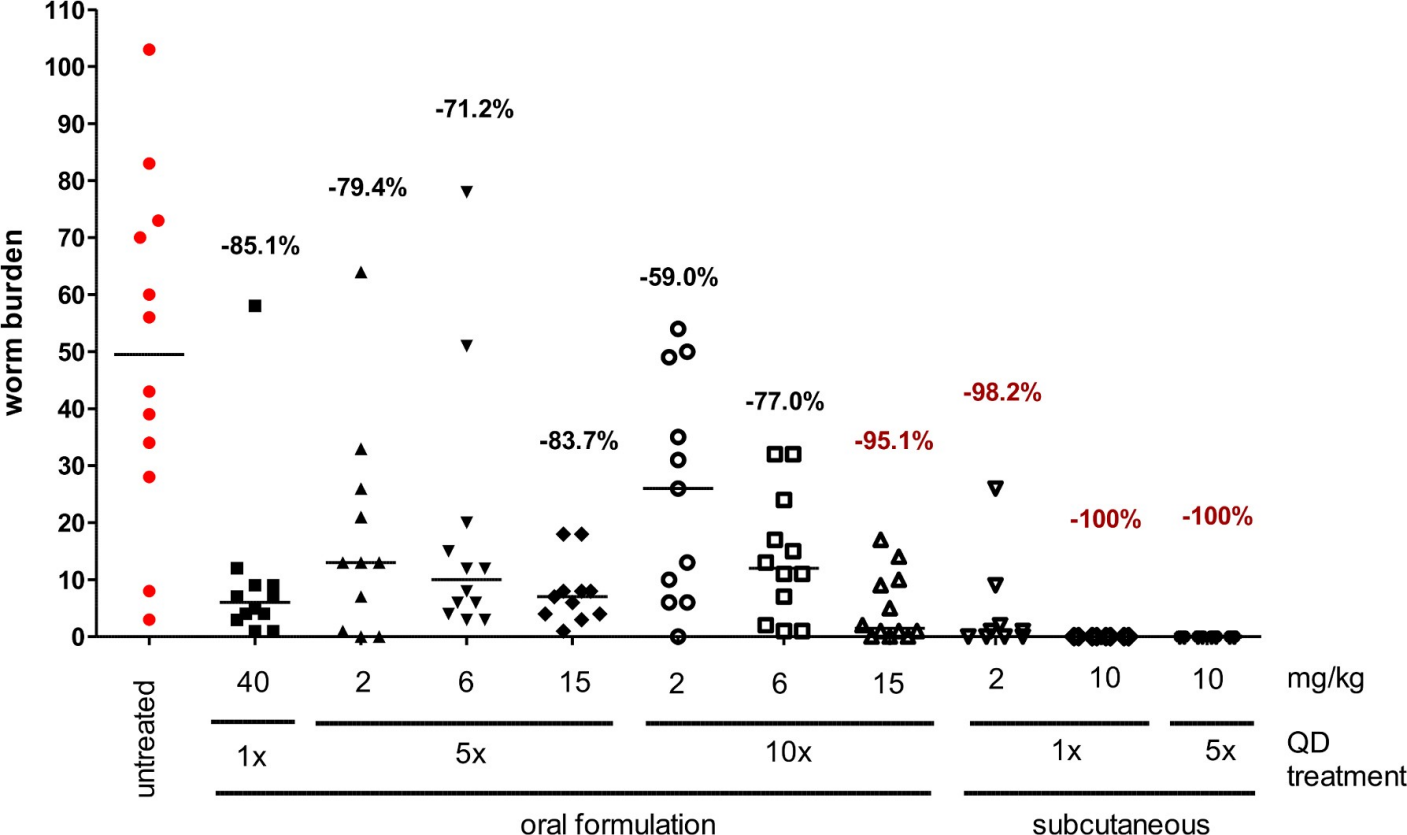
Oral and subcutaneous flubendazole treatment reduces *L*. *sigmodontis* adult worm burden in a dose and treatment duration dependent manner. *L*. *sigmodontis* adult worm burden 8 weeks post treatment end of jirds receiving oral gavages of flubendazole once (40mg/kg) or for five or ten consecutive days at 2, 6 or 15mg/kg or subcutaneous flubendazole injections once with 2 or 10mg/kg or for five 5 days (10mg/kg). Shown are medians and the reduction of adult worm burden in comparison to untreated jirds calculated from geometric means.

The primary efficacy parameter in this study was the number of intact adult *L*. *sigmodontis* worms present at the time of necropsy for each of the different FBZ treatment regimens. Eight weeks after the end of treatment all adult worms present were isolated and counted: the total worm burden is shown in Fig 1 and results are summarized in S2 Table.

All untreated animals harbored adult worms at the time of necropsy (range: 3–103 adult worms; median: 50 adult worms). One single oral treatment with 40mg/kg FBZ resulted in the statistical significant reduction of the adult worm burden by 85% (based on geometric mean, median: 88%). Five oral treatments with 2, 6, and 15mg/kg FBZ reduced the adult worm burden by 79, 71 and 84% (median: 74%, 80% and 86%), respectively. Ten-day oral treatments with 2 and 6mg/kg FBZ reduced the adult worm burden by 59 and 77% (median: 47% and 76%), respectively. 10 oral treatments with 15mg/kg reduced the adult worm burden by 95% (median: 97%). One single subcutaneous treatment with 2mg/kg FBZ reduced the adult worm burden by 98% (median: 100%). Subcutaneous treatments with 10mg/kg FBZ for 1 or 5 days resulted in a complete removal of all adult worms from all jirds.

The statistical comparison based on Dunn’s test indicates that all the subcutaneous groups (p-value < 0.0001), ten-day oral treatments with 15mg/kg (p-value <0.01), single dose with 40mg/kg (p-value <0.05) are significantly different from the untreated group (S2 Table). All other treatment groups are not significantly different from the untreated group (S2 Table).

In summary, 10 days of oral treatment with 15mg/kg and all subcutaneous administrations tested achieved greater than 90% reduction in adult worm burden, confirming a prominent macrofilaricidal effect of oral and subcutaneous FBZ treatment regimens.

### Single subcutaneous treatment with 2mg/kg FBZ completely inhibits filarial embryogenesis

**Fig 2:**
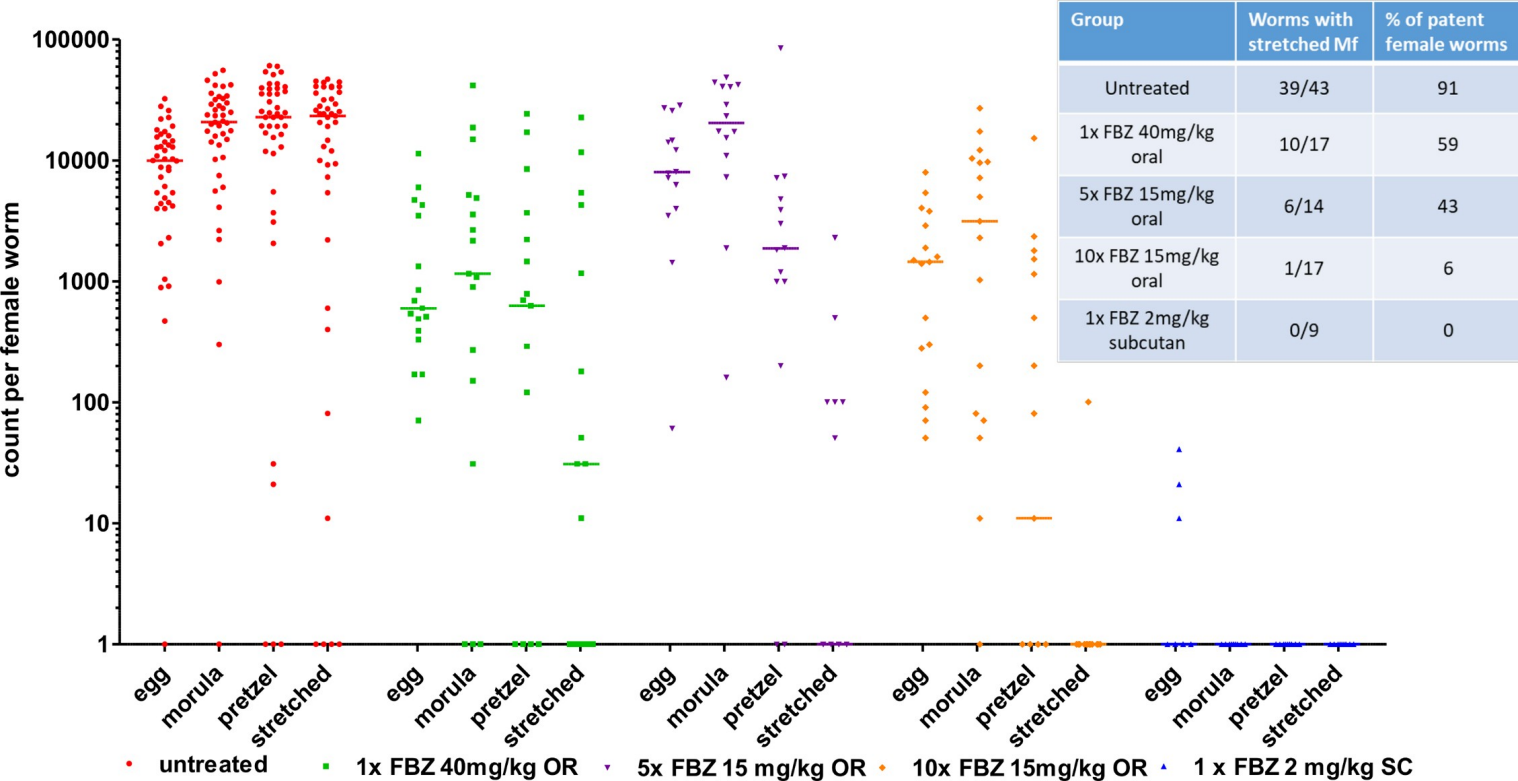
Single subcutaneous flubendazole treatment inhibits filarial embryogenesis. Shown are total number of eggs, morulae, pretzel and stretched microfilariae per female worm of jirds that were either untreated, received one oral treatment of 40mg/kg FBZ, five or ten oral treatments with 15mg/kg or one subcutaneous treatment with 2mg/kg FBZ. Shown are medians. The included table highlights the number of female adult worms harboring stretched microfilariae and the resulting frequency of patent female worms.

In order to assess whether FBZ treatment inhibited the embryogenesis and sterilized the remaining female adult worms that were not killed, embryograms were performed and the embryonal stages within the uteri of the female adult worms were enumerated, differentiated as eggs, morulae, pretzel, and stretched MF (Fig 2). The majority (91%) of female adult worms from the untreated group contained stretched MF and comparable numbers of all embryonal stages, i.e. eggs, morulae, pretzel stages. Single oral treatment with 40mg/kg FBZ reduced the number of all embryonal stages analyzed by 86.3–99.3% (based on geometric mean) and 59% of the female worms contained stretched MF. 5 and 10 oral treatments with 15mg/kg FBZ reduced the late embryonal stages (pretzel: 89.0 and 99.7%, respectively; stretched MF: 99.8 and 99.99%, respectively), resulting in 43% and 6% of female worms containing stretched MF, respectively. Single subcutaneous treatment with 2mg/kg FBZ completely inhibited the embryogenesis in all studied animals, with 3 out of 9 female worms showing low numbers of unfertilized eggs, but no fertilized embryonal stages. The other 6 female worms of the subcutaneous group completely lacked any embryonal stages. These data indicate that FBZ treatment inhibits the embryogenesis of the female adult worms.

### Blood MF levels are reduced in a dose and treatment duration dependent manner by oral FBZ treatment and completely cleared by subcutaneous FBZ treatment regimens

**Fig 3:**
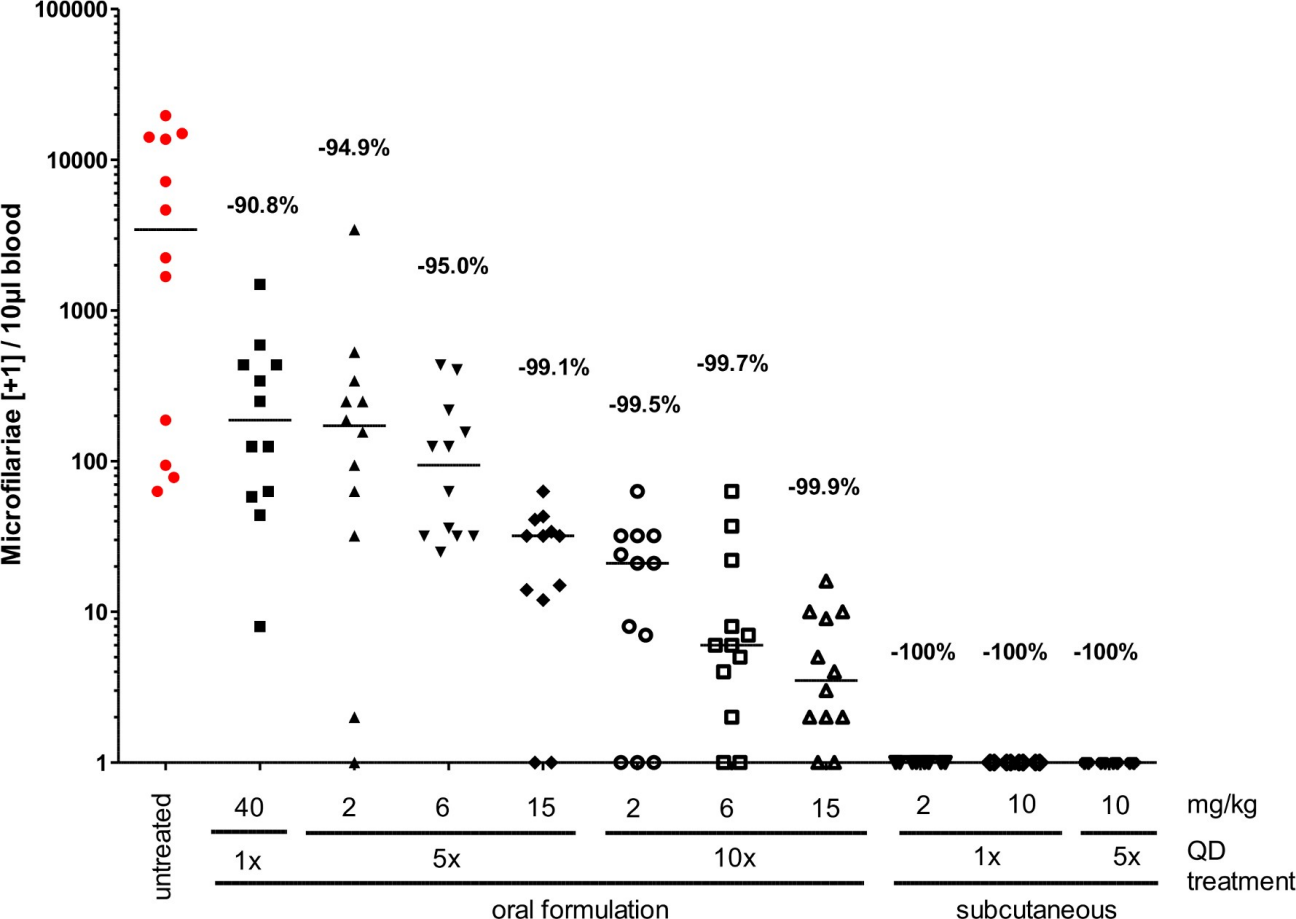
Oral and subcutaneous flubendazole treatment reduces *L*. *sigmodontis* microfilariae in a dose and treatment duration dependent manner. *L*. *sigmodontis* microfilariae counts per 10μl peripheral blood at 8 weeks post treatment end. Jirds received oral gavages of flubendazole once (40mg/kg) or for five or ten consecutive days at 2, 6 or 15mg/kg or subcutaneous flubendazole injections once with 2 or 10mg/kg or for five 5 days (10mg/kg). Shown are medians and the reduction of microfilariae levels in comparison to untreated jirds calculated from geometric means.

As FBZ treatment regimens inhibited filarial embryogenesis, we next quantified MF in the peripheral blood at the time of necropsy. All untreated jirds were MF positive at the time point of necropsy, harboring 1699 MF (geometric mean; range: 62–19.592) per 10μl of peripheral blood (Fig 3; S3 Table). The number of peripheral MF was reduced in a duration and concentration dependent manner after FBZ treatment (Fig 3; S3 Table). Single oral treatment with 40mg/kg FBZ reduced the MF load by 90.8%, 5 oral treatments with 2, 6, and 15mg/kg FBZ reduced the peripheral MF numbers by 94.9%, 95.0% and 99.1%, respectively. 10 oral treatments with 2, 6, and 15mg/kg FBZ resulted in a reduction of MF by 99.5%, 99.7% and 99.9%, respectively. All subcutaneous FBZ treatment regimens cleared the microfilaremia in all animals studied by 100%. 5 oral treatments with 15mg/kg FBZ cleared the microfilaremia in one out of 11 jirds studied and 10 oral treatments with 2, 6, and 15mg/kg FBZ cleared the microfilaremia in 2 out of 11 (2mg/kg FBZ) and 2 out of 12 animals (6 and 12mg/kg FBZ) studied (Fig 3; S3 Table).

In summary, all FBZ treatment regimens reduced the peripheral microfilaremia by more than 90%. Oral administration at the high dose (15mg/kg FBZ) for 5 days as well as prolonged treatment times with all concentrations tested (2, 6, and 15mg/kg FBZ) for 10 days reduced the peripheral microfilaremia by more than 99%, but completely cleared the MF in less than 20% of tested animals. All subcutaneous FBZ treatment regimens tested were most successful and mediated a complete clearance of the peripheral microfilaremia.

### FBZ treatment causes pathology in female adult worms and uterine microfilariae

**Fig 4:**
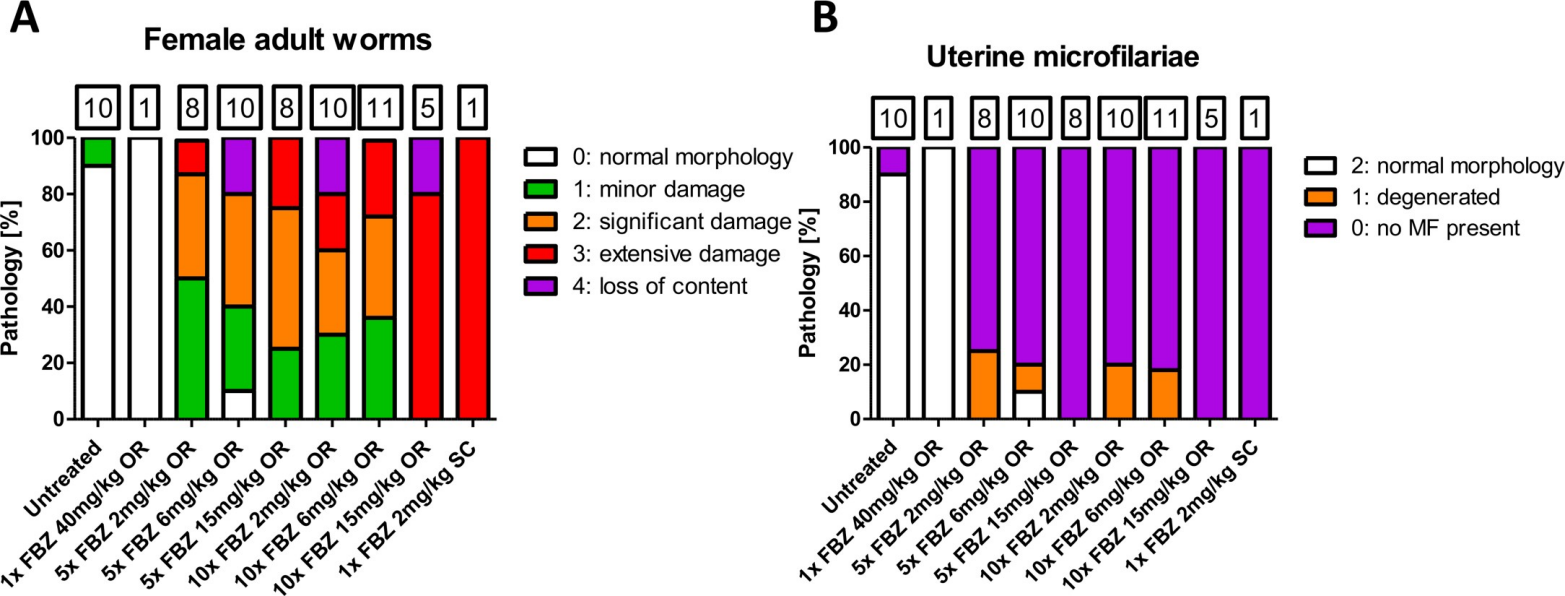
Flubendazole treatment causes pathology in female adult worms and uterine microfilariae. A, shown is the frequency of female adult worms presenting normal morphology (score 0, white bar), minor damage (score 1, green bar), significant damage (score 2, orange bar), extensive damage (score 3, red bar), or loss of content (score 4, purple bar). B, frequency of adult worms presenting no uterine microfilariae (score 0, purple bar), degenerated microfilariae (score 1, orange bar) or microfilariae with normal morphology (score 2, white bar) within the uteri. Adult worms were isolated 8 weeks post treatment end of jirds receiving oral gavages (OR) of flubendazole once (40mg/kg) or for five or ten consecutive days at 2, 6 or 15mg/kg or subcutaneous (SC) flubendazole injections once with 2mg/kg. The total number of female adult worms analyzed per group is indicated above each stacked bar.

**Fig 5:**
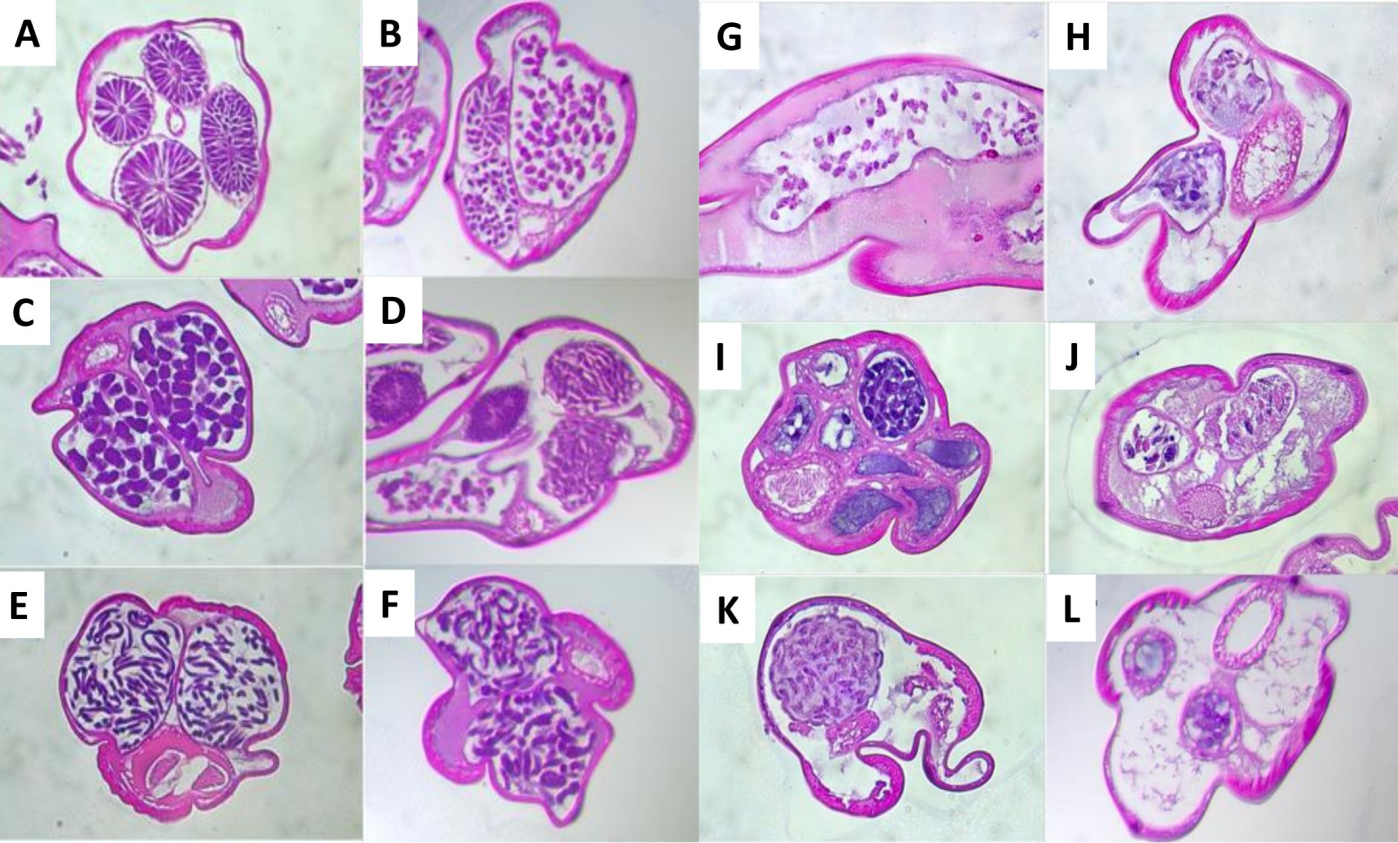
Comparison of morphologies observed in healthy female adult worms and female adult worms isolated of flubendazole treated jirds. Morphologies seen that were defined as being within normal range (i.e. scored as 0) are displayed in A-F. A, ovarian organs; B, early normal developmental forms—oocytes; C, normal morula forms; D, various stages including embryos; E, fully developed microfilariae beginning to extend. F, developed microfilariae in egg shells, including a normal intestine. Examples of degenerative morphological changes seen in the adult *Litomosoides* examined are shown in G-L. G, degenerating oocytes; H, early calcification in an oocyte filled uterine horn (the lower uterine segment); I, calcification of uterus; J, degenerated early morula forms; K, blocked uterus filled with calcified mass containing various embedded parasitic forms; L, distended female worm segment with two uterine sections nudging calcification.

As a permanent sterilization prevents the transmission of filarial disease, we next investigated the impact of FBZ treatment on filarial pathology in selected treatment groups. Oral treatment regimens for five days with 6 and 15mg/kg or 10 days with 2 and 6mg/kg FBZ resulted in a comparable pathological score with an average of 2 indicating a significant pathology of the female adult worms (Fig 4A, Fig 5). 5x oral treatments with 2mg/kg FBZ caused an average pathological score of 1.5 in the analyzed female adult worms indicating a minor to significant damage of the adult worms. Adult female worms isolated from animals treated for 10 days with 15mg/kg orally had all extensive damage and one out of 5 worms lost its contents (highest pathological score). Only one single worm was available for pathological analysis of each the single oral and single subcutaneous treatment groups, revealing no pathology and extensive damage, respectively. As expected, all female worms isolated from untreated animals had a normal morphology, except one single worm with minor pathology. Changes in the body wall of the remaining adult worms were varied with the most significant damage seen in those worms that had considerable damage in their other organs; this was also the case with the intestine where the most damage was seen in those worms that had damage to their other organs. The major degenerative changes (e.g. vacuolation, membrane disruption, breakdown of anatomical structure and calcification) seen were in the uterus and with the uterine contents (developing MF); the major change in the majority of adult female worms was an interruption in the development of the uterine forms of MF particularly at the morulae stage (Fig 5), with stages beyond the morula form also degenerating or undergoing calcification. Earlier developmental forms (e.g. oocytes) when present were either sparse in number or were abnormally vacuolated and also appeared to be undergoing degeneration. Fully formed MF still present within the uterus also were abnormal and appeared to be degenerating in all animals that received repeated oral FBZ doses or subcutaneous FBZ treatment (Fig 4B, Fig 5). These data support the findings from the embyrograms, indicating an inhibition of embryogenesis caused by oral treatment with 15mg/kg for 10 days and treatments via the subcutaneous route, which in all likelihood is permanent and signals the demise of the adult female worms.

### Subcutaneous FBZ administration leads to a slow FBZ release with constant plasma levels while orally administered FBZ is rapidly absorbed and cleared

Using pharmacokinetic analysis we compared C_max_ and AUC values of FBZ between the different treatment regimens and route of administrations. The pharmacokinetic parameters were also calculated for its main metabolites hydrolyzed FBZ (H-FBZ) and reduced FBZ (R-FBZ).

After single or repeated oral administration of FBZ to female jirds at 2, 6, and 15mg/kg/day for 5 or 10 days or 40mg/kg after single dose, peak plasma concentrations were observed between 0.5 and 1h after dosing, suggesting a rapid absorption (Table 1, S1A Fig). Mean C_max_ and AUC_0-24h_ values increased roughly dose proportionally between 2mg/kg/day up to 40mg/kg (Table 1, S1A Fig). No changes in C_max_ and AUC_0-24h_ values were observed with longer duration of dosing with values similar between day 5 and day 10 (Table 1, S1A Fig). The R-FBZ/FBZ ratio ranged from 0.008 to 0.027 and from 0.29 to 0.59 for H-FBZ/FBZ across all FBZ dosed groups (Table 1).

**Table 1:** Rapid plasma clearance of orally administered flubendazole and slow flubendazole release with constant plasma levels of subcutaneously administered flubendazole. Shown are the highest observed flubendazole plasma concentration (C_max_), the time point of the highest observed flubendazole plasma concentration (t_max_), the area under the curve (total drug exposure in blood plasma against time, AUC_0-24h_), the AUC ratio of the hydrolyzed flubendazole to flubendazole (H-FBZ/FBZ) and the reduced flubendazole to flubendazole (R-FBZ/FBZ) of the different treatment groups. Data was obtained using a sparse sampling approach with 3–4 animals per group and time point.

**After Oral Administration**						
**Day**	**Doses (mg/kg)**	**C**_**max**_ **(**μ**g/ml)**	**t**_**max**_ **(h)**	**AUC**_**0-24h**_ **(**μ**g.h/ml)**	**H-FBZ /FBZ AUC ratio**	**R-FBZ/FBZ AUC ratio**
1	40	8.8	1	52	0.3	0.02
5	2	0.6	1	2.8	0.5	0.01
5	6	1.6	0.5	7.8	0.6	0.01
5	15	4.4	1	21	0.4	0.02
10	2	0.6	0.5	2.5	0.3	0.01
10	6	1.8	0.5	8.9	0.4	0.01
10	15	3.1	1	20	0.3	0.03
**After SC administration**						
**Day**	**Doses (mg/kg)**	**C**_**max**_ **(**μ**g/ml)**	**t**_**max**_ **(h)**	**AUC**_**0-Day52 or Day57**_ **(**μ**g.h/ml)**	**H-FBZ /FBZ AUC ratio**	**R-FBZ/FBZ AUC ratio**
1	2	0.160	48	9.8	0.02	0.0001
1	10	0.167	48	15	n.d.	0.009
5	10	0.05	1	31	0.35	0.04

After subcutaneous administration, FBZ was slowly released from the injection site and plasma levels remained constant from day 5 up to necropsy (Table 1, S1B Fig). At 2 and 10mg/kg single dose, the C_max_ was similar and AUC_0- Days 57_ increased less than dose proportionally between the 2 doses (Table 1, S1B Fig). After comparison of 10mg/kg single dose and after 5 days of administration, no increase in C_max_ values was seen and exposure (AUC_0-Day52_) increased by two (Table 1, S1B Fig). In this study the R-FBZ/FBZ ratio ranged from 0.0001 to 0.040 and from 0.02 to 0.35 for H-FBZ/FBZ across all subcutaneous administered FBZ dosed groups (Table 1).

The plasma profiles were completely different between oral and subcutaneous routes. After oral administration FBZ was rapidly cleared with a concentration of 496ng/ml at 24h in the 40mg/kg single dose group and 89-116ng/ml after 15mg/kg/day at day 5 or day 10 (S1A Fig). After subcutaneous administration at 10mg/kg/day for 5 days, the concentrations were approximately 45ng/ml at 24h after the last dosing decreasing up to 16ng/ml at day 57 (S1B Fig). The AUC_0-120h_ was 14% of the AUC_0-d57_ (S1B Fig). The R-FBZ/FBZ and H-FBZ/FBZ ratios were similar between the two routes of administration except for 1x2mg subcutaneous FBZ treatment, which resulted in a lower ratio (Table 1).

## Discussion

With the recent advances in the elimination efforts for lymphatic filariasis using the triple therapy, it is believed that the elimination will be essentially accelerated given a moderate to high coverage [6]. Similarly, in the Americas and most endemic countries in Africa a shift from onchocerciasis control to elimination was achieved [9].

Identification of a short-course macrofilaricidal drug would be an essential tool for the treatment of the remaining infected individuals, reducing program time frames required to eliminate onchocerciasis and lymphatic filariasis given a sufficient drug coverage and provide treatment options in areas with suboptimal ivermectin responses [11, 12].

In the present study we demonstrate that oral as well as subcutaneous FBZ administrations provide such a macrofilaricidal efficacy in the *L*. *sigmodontis* jird model. Oral treatments resulted in a dose and treatment duration-dependent reduction of the adult worm burden by 8 weeks post treatment end with 10 days of oral FBZ treatment with 15mg/kg achieving a reduction in the adult worm burden by 95% and a cure rate of 25%. Subcutaneous administration of FBZ was even more effective leading to a complete clearance of the adult worm burden in all animals studied that received single or 5 days treatments with 10mg/kg, as well as to a 98.2% reduction in the adult worm burden and a cure rate of 58% in animals receiving a single subcutaneous administration of 2mg/kg. This was in line with previous studies demonstrating a macrofilaricidal effect after subcutaneous FBZ treatments in different filarial rodent models [16–18].

Importantly, all remaining adult worms isolated from oral FBZ treated animals in our study showed pathological damage ranging from minor to extensive damage. This suggests that the observed clearance rate of the adult worms at 8 weeks after the end of treatment may further increase with a longer observation time. Next to the provision of a macrofilaricidal efficacy, a drug that permanently sterilizes the female adult worms would equally support the elimination of onchocerciasis by preventing the transmission of the disease. Our results demonstrate that oral FBZ treatment with 15mg/kg for 5 days significantly reduced the number of later embryonal stages within the female adult worms with 43% of analyzed female worms being patent (albeit with 27-fold reduced uterine MF counts) in comparison to 91% in the control group. Extended oral FBZ treatment with 15mg/kg for 10 days further promoted this effect with only one female adult worm out of 17 being patent. Single subcutaneous FBZ treatment with 2mg/kg completely prevented the occurrence of later embryonal stages in all female worms analyzed. Histopathological analysis of the uterine MF confirmed that all repeated oral FBZ treatment regimens reduced the presence of MF with remaining MF undergoing degeneration. Accordingly, peripheral blood MF were reduced 8 weeks after the end of treatment in a dose and treatment duration dependent manner, with all FBZ treatment regimens tested exceeding a 90% reduction in the MF levels and all tested 10-day or 5-day treatment regimens at 15mg/kg exceeding a 99% reduction in microfilaremia. However, only subcutaneous treatment regimens completely cleared peripheral microfilaremia in all animals studied.

These observations indicate that FBZ has a macrofilaricidal efficacy and reduces peripheral MF by impairing filarial embryogenesis through the induction of pathology within the filarial uteri. The lack of a strong direct microfilaricidal effect is shown by the study of Sjoberg et al., which is part of this FBZ PLOS NTD collection, and has the advantage that this should reduce inflammatory reactions in response to dying MF (Mazzotti reaction), which, as in the case of historic DEC treatment of onchocerciasis, lead to blindness or severe dermatitis [3]. Furthermore, lack of a direct microfilaricidal effect by FBZ may further allow the usage of FBZ in areas co-endemic for *Loa*, which are currently excluded from MDA programs due to the risk of *Loa loa* patients with high MF counts to develop life threatening severe adverse events following treatment. Such a lack of microfilaricidal activity against *Loa loa* by FBZ was recently supported by *in vitro* studies [34].

Our histopathological analyses of female adult worms further showed that repeated oral doses of FBZ at all concentrations tested induced minor to extensive damage in the remaining female adult worms and 10 days of oral treatment with 15mg/kg resulted in extensive damage and in part the loss of content in all remaining female worms. Such a damage of the reproductive tissues and hypodermis of filarial nematodes by FBZ exposure was previously described for *in vitro* studies using *Brugia malayi* female worms [35, 37, 38].

In the past, orally administered FBZ had a poor bioavailability [39], which was improved by the tested newly developed oral formulation used in this study. Nevertheless, the comparison of the efficacy of orally and subcutaneously administered FBZ clearly demonstrates that subcutaneous administrations were more efficacious by clearing all adult worms when administered at 10mg/kg once.

Pharmacokinetic analysis in our study confirm that the reduction of adult worm burden, and also damage to the surviving worms and thus, sterility, is mainly driven by the long-term exposure to FBZ. All three subcutaneous FBZ treatment regimens led to a slow FBZ release from the injection site and the plasma levels remained constant from day 5 up to the study endpoint 8 weeks after treatment end. In contrast, orally administered FBZ was rapidly cleared from plasma and FBZ plasma concentrations peaked 0.5-1h after gavage with a dose-proportional increase. Extended oral FBZ administration from day 5 to day 10 did not change C_max_ and AUC_0-24h_ values and three subcutaneous FBZ treatment regimens resulted in similar C_max_ values which were 4 to 26-fold lower than after repeated oral administration with 2 or 15mg/kg, respectively. Therefore, reduction of adult worm burden is mainly driven by the long-term exposure to FBZ. However, oral treatment regimens in this study demonstrate that high C_max_ values result in a significant reduction of adult worm burden as well, which achieved 95% efficacy in adult worm reduction when administered at 15mg/kg for 10 days.

In summary, this study indicates the potent macrofilaricidal effects of FBZ in *L*. *sigmodontis*-infected jirds using repeated oral or a single parenteral administration. Histopathological analysis confirmed that FBZ treatment causes degeneration of filarial uterine tissue, preventing filarial embryogenesis and the release of MF.

## Supporting information

S1 Fig Orally administered flubendazole is rapidly cleared from plasma while constant plasma levels are obtained following subcutaneous flubendazole administration. Mean (n = 4 per time point) plasma (ng/ml) concentrations of flubendazole after the last day of dosing after single and repeated (A) oral or (B) subcutaneous treatment. Jirds received oral gavages of flubendazole once (40mg/kg) or for five or ten consecutive days at 2, 6 or 15mg/kg or subcutaneous flubendazole injections once with 2 or 10mg/kg or for five 5 days (10mg/kg).
(TIF)

S1 Table Overview of experimental groups and treatment regimens. (DOCX)

S2 Table Detailed overview of the adult worm burden 8 weeks post treatment start. Individual adult worm counts, arithmetic, geometric mean as well as median of the adult worm counts, calculated efficacy of worm reduction, p-values, and number of animals without adult worms. Jirds received oral gavages of flubendazole once (40mg/kg) or for five or ten consecutive days at 2, 6 or 15mg/kg or subcutaneous flubendazole injections once with 2 or 10mg/kg or for five 5 days (10mg/kg).
(DOCX)

S3 Table Detailed overview of the peripheral blood microfilariae counts 8 weeks post treatment start. Arithmetic, geometric mean as well as median of the peripheral blood microfilariae (MF) counts, calculated efficacy of MF reduction in comparison to untreated controls, number and frequency of animals without peripheral microfilaremia. Jirds received oral gavages of flubendazole once (40mg/kg) or for five or ten consecutive days at 2, 6 or 15mg/kg or subcutaneous flubendazole injections once with 2 or 10mg/kg or for five 5 days (10mg/kg).
(DOCX)

## References

[1] Murray CJ, Vos T, Lozano R, Naghavi M, Flaxman AD, Michaud C, et al Disability-adjusted life years (DALYs) for 291 diseases and injuries in 21 regions, 1990–2010: a systematic analysis for the Global Burden of Disease Study 2010. Lancet. 2012;380(9859):2197–223. Epub 2012/12/19. 10.1016/S0140-6736(12)61689-4 . 23245608

[2] Taylor MJ, Hoerauf A, Bockarie M. Lymphatic filariasis and onchocerciasis. Lancet. 2010;376(9747):1175–85. Epub 2010/08/27. S0140-6736(10)60586-7 [pii] 10.1016/S0140-6736(10)60586-7 . 20739055

[3] Tamarozzi F, Halliday A, Gentil K, Hoerauf A, Pearlman E, Taylor MJ. Onchocerciasis: the role of Wolbachia bacterial endosymbionts in parasite biology, disease pathogenesis, and treatment. Clin Microbiol Rev. 2011;24(3):459–68. Epub 2011/07/08. 24/3/459 [pii] 10.1128/CMR.00057-10 21734243 PMC3131055

[4] Hoerauf A, Pfarr K, Mand S, Debrah AY, Specht S. Filariasis in Africa—treatment challenges and prospects. Clin Microbiol Infect. 2011;17(7):977–85. Epub 2011/07/05. 10.1111/j.1469-0691.2011.03586.x . 21722251

[5] Hoerauf A. Filariasis: new drugs and new opportunities for lymphatic filariasis and onchocerciasis. Curr Opin Infect Dis. 2008;21(6):673–81. Epub 2008/11/04. 10.1097/QCO.0b013e328315cde7 [doi]00001432-200812000-00015 [pii]. . 18978537

[6] Irvine MA, Stolk WA, Smith ME, Subramanian S, Singh BK, Weil GJ, et al Effectiveness of a triple-drug regimen for global elimination of lymphatic filariasis: a modelling study. Lancet Infect Dis. 2017;17(4):451–8. Epub 2016/12/26. 10.1016/S1473-3099(16)30467-4 . 28012943

[7] Thomsen EK, Sanuku N, Baea M, Satofan S, Maki E, Lombore B, et al Efficacy, Safety, and Pharmacokinetics of Coadministered Diethylcarbamazine, Albendazole, and Ivermectin for Treatment of Bancroftian Filariasis. Clin Infect Dis. 2015 Epub 2015/10/22. 10.1093/cid/civ882 . 26486704

[8] WHO. Progress toward eliminating onchocerciasis in the WHO Region of the Americas: verification of elimination of transmission in Mexico. Wkly Epidemiol Rec. 2015;90(43):577–81. 26495516

[9] WHO. Progress report on the elimination of human onchocerciasis, 2016–2017. Wkly Epidemiol Rec. 2017;92(45):681–700. 29130679

[10] Awadzi K, Boakye DA, Edwards G, Opoku NO, Attah SK, Osei-Atweneboana MY, et al An investigation of persistent microfilaridermias despite multiple treatments with ivermectin, in two onchocerciasis-endemic foci in Ghana. Ann Trop Med Parasitol. 2004;98(3):231–49. Epub 2004/05/04. 10.1179/000349804225003253 . 15119969

[11] Dunn C, Callahan K, Katabarwa M, Richards F, Hopkins D, Withers PC Jr., et al The Contributions of Onchocerciasis Control and Elimination Programs toward the Achievement of the Millennium Development Goals. PLoS Negl Trop Dis. 2015;9(5):e0003703 Epub 2015/05/23. 10.1371/journal.pntd.0003703 25996946 PMC4440802

[12] Geary TG, Mackenzie CD. Progress and challenges in the discovery of macrofilaricidal drugs. Expert Rev Anti Infect Ther. 2011;9(8):681–95. Epub 2011/08/09. 10.1586/eri.11.76 . 21819332

[13] Horton RJ. Benzimidazoles in a wormy world. Parasitol Today. 1990;6(4):106 Epub 1990/04/01. . 15463310 10.1016/0169-4758(90)90225-s

[14] Yangco BG, Klein TW, Deresinski SC, Vickery AC, Craig CP. Flubendazole and mebendazole in the treatment of trichuriasis and other helminthiases. Clinical therapeutics. 1981;4(4):285–90. Epub 1981/01/01. . 7332916

[15] Kan SP. The anthelmintic effects of flubendazole on Trichuris trichiura and Ascaris lumbricoides. Trans R Soc Trop Med Hyg. 1983;77(5):668–70. Epub 1983/01/01. . 6659046 10.1016/0035-9203(83)90199-2

[16] Mackenzie CD, Geary TG. Flubendazole: a candidate macrofilaricide for lymphatic filariasis and onchocerciasis field programs. Expert Rev Anti Infect Ther. 2011;9(5):497–501. Epub 2011/05/26. 10.1586/eri.11.30 . 21609260

[17] Denham DA, Samad R, Cho SY, Suswillo RR, Skippins SC. The anthelmintic effects of flubendazole on Brugia pahangi. Trans R Soc Trop Med Hyg. 1979;73(6):673–6. Epub 1979/01/01. . 538808 10.1016/0035-9203(79)90018-x

[18] Mak JW. Antifilarial activity of mebendazole and flubendazole on Breinlia booliati. Trans R Soc Trop Med Hyg. 1981;75(2):306–7. Epub 1981/01/01. . 7303142 10.1016/0035-9203(81)90343-6

[19] Dominguez-Vazquez A, Taylor HR, Greene BM, Ruvalcaba-Macias AM, Rivas-Alcala AR, Murphy RP, et al Comparison of flubendazole and diethylcarbamazine in treatment of onchocerciasis. Lancet. 1983;1(8317):139–43. Epub 1983/01/22. . 6130195 10.1016/s0140-6736(83)92753-8

[20] Morris CP, Evans H, Larsen SE, Mitre E. A comprehensive, model-based review of vaccine and repeat infection trials for filariasis. Clin Microbiol Rev. 2013;26(3):381–421. Epub 2013/07/05. 26/3/381 [pii] 10.1128/CMR.00002-13 . 23824365 PMC3719488

[21] Allen JE, Adjei O, Bain O, Hoerauf A, Hoffmann WH, Makepeace BL, et al Of mice, cattle, and humans: the immunology and treatment of river blindness. PLoS Negl Trop Dis. 2008;2(4):e217 10.1371/journal.pntd.0000217 . 18446236 PMC2323618

[22] Volkmann L, Fischer K, Taylor M, Hoerauf A. Antibiotic therapy in murine filariasis (Litomosoides sigmodontis): comparative effects of doxycycline and rifampicin on Wolbachia and filarial viability. Trop Med Int Health. 2003;8(5):392–401. Epub 2003/05/20. doi: 1040 [pii]. . 12753632 10.1046/j.1365-3156.2003.01040.x

[23] Specht S, Pfarr KM, Arriens S, Hubner MP, Klarmann-Schulz U, Koschel M, et al Combinations of registered drugs reduce treatment times required to deplete Wolbachia in the Litomosoides sigmodontis mouse model. PLoS Negl Trop Dis. 2018;12(1):e0006116 Epub 2018/01/05. 10.1371/journal.pntd.0006116 . 29300732 PMC5771630

[24] Hoerauf A, Nissen-Pahle K, Schmetz C, Henkle-Duhrsen K, Blaxter ML, Buttner DW, et al Tetracycline therapy targets intracellular bacteria in the filarial nematode Litomosoides sigmodontis and results in filarial infertility. J Clin Invest. 1999;103(1):11–8. Epub 1999/01/12. 10.1172/JCI4768 9884329 PMC407866

[25] Taylor MJ, Makunde WH, McGarry HF, Turner JD, Mand S, Hoerauf A. Macrofilaricidal activity after doxycycline treatment of Wuchereria bancrofti: a double-blind, randomised placebo-controlled trial. Lancet. 2005;365(9477):2116–21. Epub 2005/06/21. S0140-6736(05)66591-9 [pii] 10.1016/S0140-6736(05)66591-9 . 15964448

[26] Hoerauf A, Mand S, Adjei O, Fleischer B, Buttner DW. Depletion of wolbachia endobacteria in Onchocerca volvulus by doxycycline and microfilaridermia after ivermectin treatment. Lancet. 2001;357(9266):1415–6. Epub 2001/05/18. S0140-6736(00)04581-5 [pii] 10.1016/S0140-6736(00)04581-5 . 11356444

[27] Debrah AY, Specht S, Klarmann-Schulz U, Batsa L, Mand S, Marfo-Debrekyei Y, et al Doxycycline Leads to Sterility and Enhanced Killing of Female Onchocerca volvulus Worms in an Area With Persistent Microfilaridermia After Repeated Ivermectin Treatment: A Randomized, Placebo-Controlled, Double-Blind Trial. Clin Infect Dis. 2015;61(4):517–26. Epub 2015/05/08. 10.1093/cid/civ363 25948064 PMC4518165

[28] Reddy AB, Rao UR, Chandrashekar R, Shrivastava R, Subrahmanyam D. Comparative efficacy of some benzimidazoles and amoscanate (Go.9333) against experimental filarial infections. Tropenmedizin und Parasitologie. 1983;34(4):259–62. Epub 1983/12/01. . 6665868

[29] Zahner H, Schares G. Experimental chemotherapy of filariasis: comparative evaluation of the efficacy of filaricidal compounds in Mastomys coucha infected with Litomosoides carinii, Acanthocheilonema viteae, Brugia malayi and B. pahangi. Acta Trop. 1993;52(4):221–66. Epub 1993/01/01. . 8094587 10.1016/0001-706x(93)90010-9

[30] Zahner H, Taubert A, Harder A, von Samson-Himmelstjerna G. Effects of Bay 44–4400, a new cyclodepsipeptide, on developing stages of filariae (Acanthocheilonema viteae, Brugia malayi, Litomosoides sigmodontis) in the rodent Mastomys coucha. Acta Trop. 2001;80(1):19–28. Epub 2001/08/10. . 11495640 10.1016/s0001-706x(01)00144-9

[31] Ajendra J, Specht S, Ziewer S, Schiefer A, Pfarr K, Parcina M, et al NOD2 dependent neutrophil recruitment is required for early protective immune responses against infectious Litomosoides sigmodontis L3 larvae. Scientific reports. 2016;6:39648 Epub 2016/12/23. 10.1038/srep39648 . 28004792 PMC5177913

[32] Ziewer S, Hübner MP, Dubben B, Hoffmann WH, Bain O, Martin C, et al Immunization with L. sigmodontis Microfilariae Reduces Peripheral Microfilaraemia after Challenge Infection by Inhibition of Filarial Embryogenesis. PLoS Negl Trop Dis. 2012;6(3):e1558 10.1371/journal.pntd.0001558 PNTD-D-11-00900 [pii]. . 22413031 PMC3295809

[33] Ajendra J, Specht S, Neumann AL, Gondorf F, Schmidt D, Gentil K, et al ST2 Deficiency Does Not Impair Type 2 Immune Responses during Chronic Filarial Infection but Leads to an Increased Microfilaremia Due to an Impaired Splenic Microfilarial Clearance. PLoS ONE. 2014;9(3):e93072 Epub 2014/03/26. 10.1371/journal.pone.0093072 . 24663956 PMC3963995

[34] O'Neill M, Njouendou JA, Dzimianski M, Burkman E, Ndongmo PC, Kengne-Ouafo JA, et al Potential Role for Flubendazole in Limiting Filariasis Transmission: Observations of Microfilarial Sensitivity. Am J Trop Med Hyg. 2017 Epub 2017/11/17. 10.4269/ajtmh.17-0390 . 29141764 PMC5928712

[35] O'Neill M, Mansour A, DiCosty U, Geary J, Dzimianski M, McCall SD, et al An In Vitro/In Vivo Model to Analyze the Effects of Flubendazole Exposure on Adult Female Brugia malayi. PLoS Negl Trop Dis. 2016;10(5):e0004698 Epub 2016/05/06. 10.1371/journal.pntd.0004698 27145083 PMC4856366

[36] Mackenzie CD, Behan‐Braman A, Hauptman J, Geary TG. Assessing the Viability and Degeneration of the Medically Important Filarial Nematodes. Nematology—Concepts, Diagnosis and Control edited by Shah M and Mahamood M. 2017 10.5772/intechopen.69512

[37] O'Neill M, Geary JF, Agnew DW, Mackenzie CD, Geary TG. In vitro flubendazole-induced damage to vital tissues in adult females of the filarial nematode Brugia malayi. Int J Parasitol Drugs Drug Resist. 2015;5(3):135–40. Epub 2015/08/20. 10.1016/j.ijpddr.2015.06.002 26288741 PMC4534755

[38] O'Neill M, Ballesteros C, Tritten L, Burkman E, Zaky WI, Xia J, et al Profiling the macrofilaricidal effects of flubendazole on adult female Brugia malayi using RNAseq. Int J Parasitol Drugs Drug Resist. 2016;6(3):288–96. Epub 2016/10/14. 10.1016/j.ijpddr.2016.09.005 27733308 PMC5196492

[39] Ceballos L, Mackenzie C, Geary T, Alvarez L, Lanusse C. Exploring the potential of flubendazole in filariasis control: evaluation of the systemic exposure for different pharmaceutical preparations. PLoS Negl Trop Dis. 2014;8(5):e2838 Epub 2014/05/31. 10.1371/journal.pntd.0002838 24874646 PMC4038472

