# Supplementary figures and images for: Macrofilaricidal efficacy of single and repeated oral and subcutaneous doses of flubendazole in Litomosoides sigmodontis infected jirds
Source: PLoS Negl Trop Dis. 2019 Jan 16;13(1):e0006320. doi: 10.1371/journal.pntd.0006320 (PMC6334906; doi:10.1371/journal.pntd.0006320)

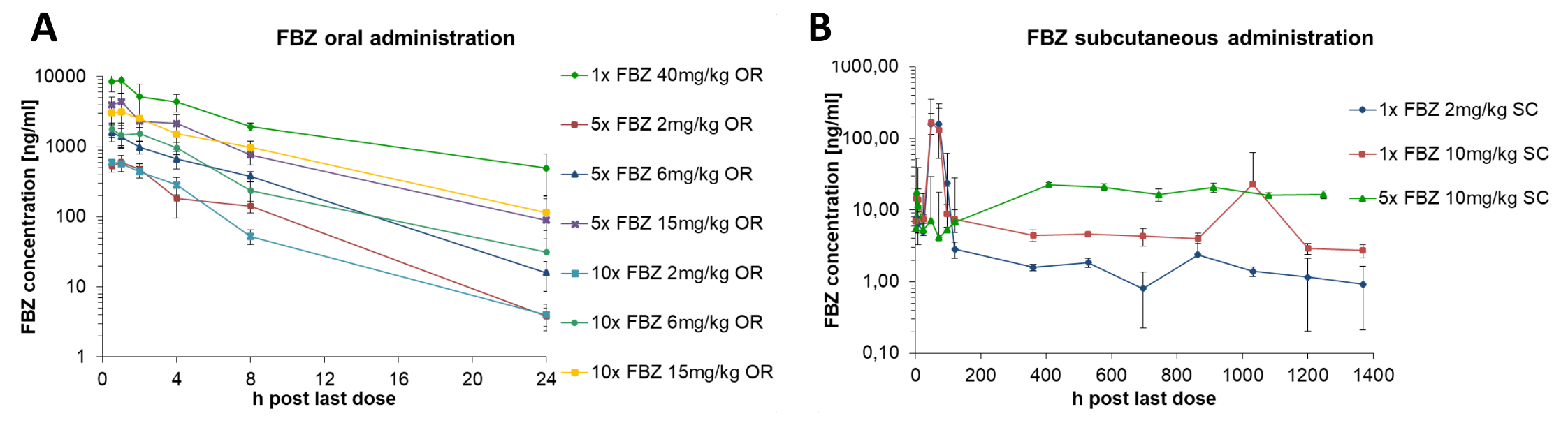

Supplement: S1 Fig — Mean (n = 4 per time point) plasma (ng/ml) concentrations of flubendazole after the last day of dosing after single and repeated (A) oral or (B) subcutaneous treatment. Jirds received oral gavages of flubendazole once (40mg/kg) or for five or ten consecutive days at 2, 6 or 15mg/kg or subcutaneous flubendazole injections once with 2 or 10mg/kg or for five 5 days (10mg/kg). (TIF) [file pntd.0006320.s001.tif]

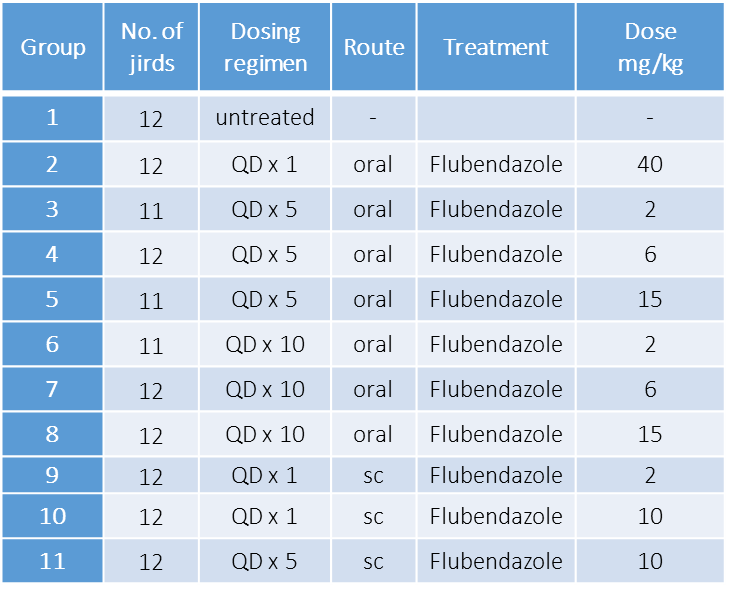

Supplement: S1 Table — (DOCX) [file pntd.0006320.s002.docx]

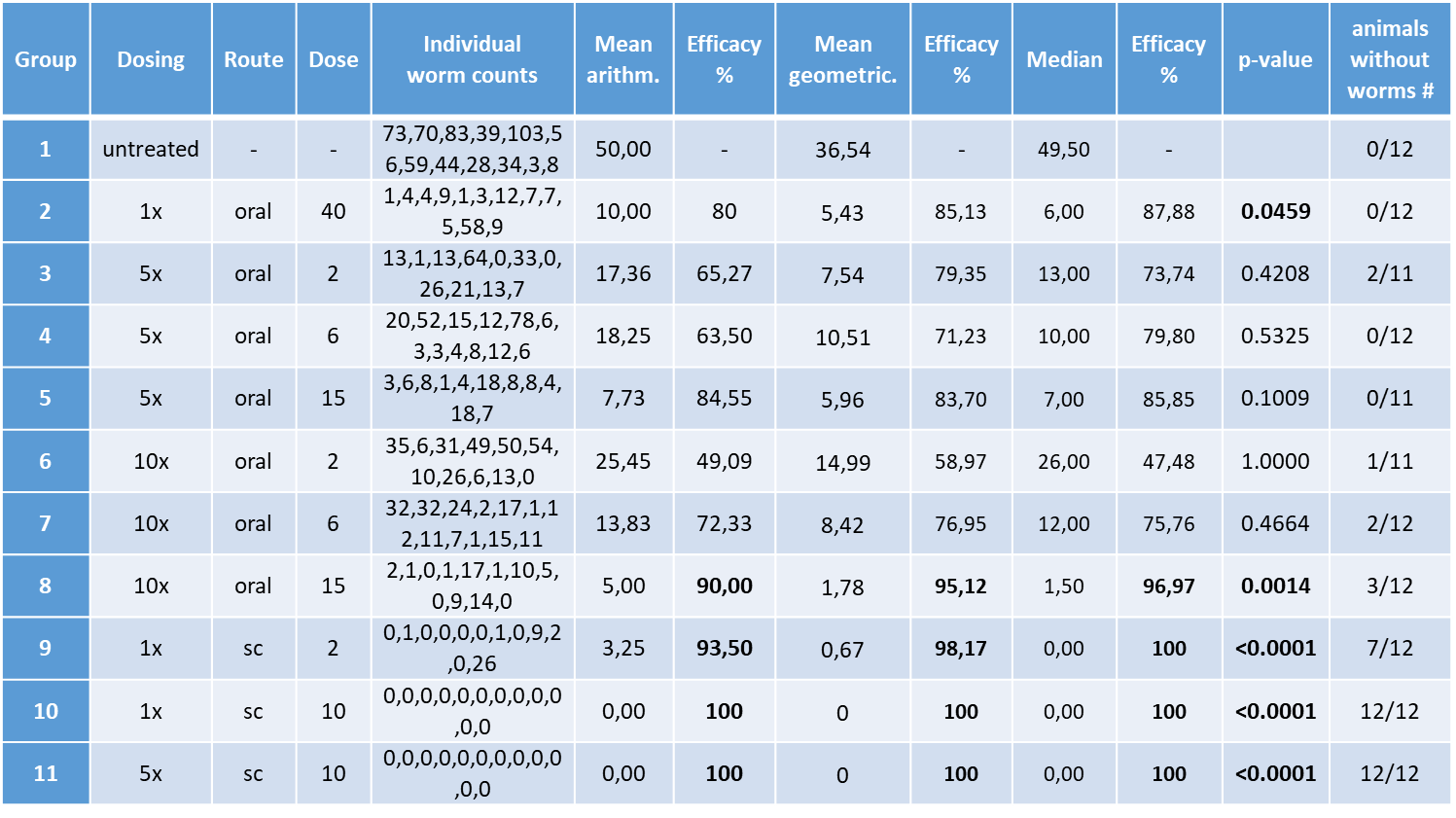

Supplement: S2 Table — Individual adult worm counts, arithmetic, geometric mean as well as median of the adult worm counts, calculated efficacy of worm reduction, p-values, and number of animals without adult worms. Jirds received oral gavages of flubendazole once (40mg/kg) or for five or ten consecutive days at 2, 6 or 15mg/kg or subcutaneous flubendazole injections once with 2 or 10mg/kg or for five 5 days (10mg/kg). (DOCX) [file pntd.0006320.s003.docx]

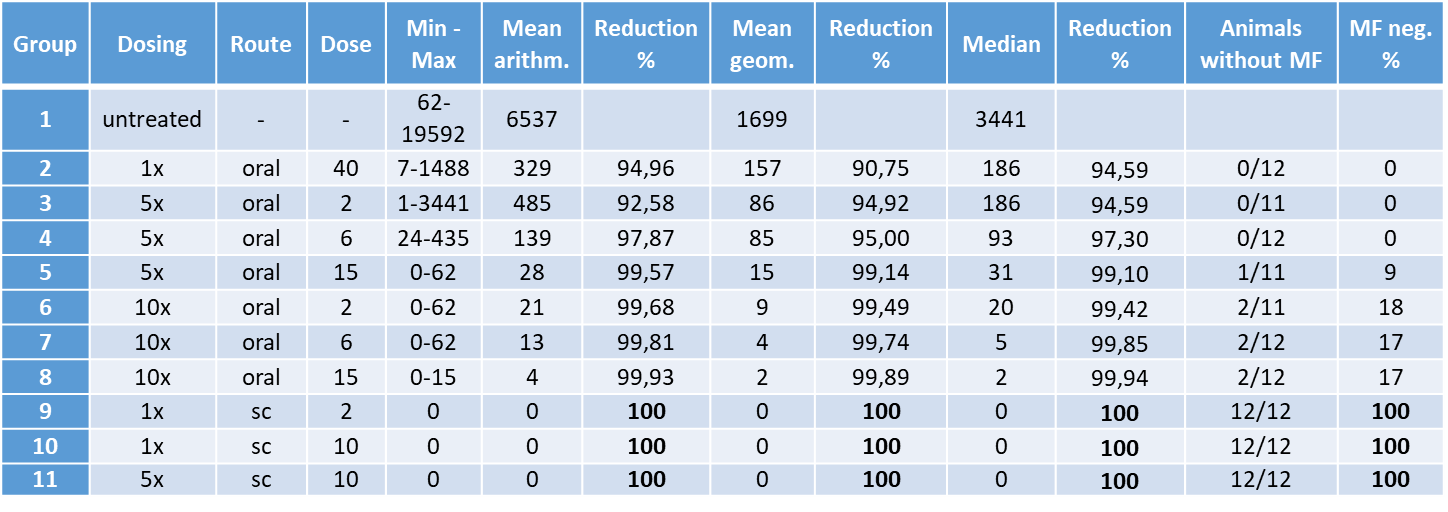

Supplement: S3 Table — Arithmetic, geometric mean as well as median of the peripheral blood microfilariae (MF) counts, calculated efficacy of MF reduction in comparison to untreated controls, number and frequency of animals without peripheral microfilaremia. Jirds received oral gavages of flubendazole once (40mg/kg) or for five or ten consecutive days at 2, 6 or 15mg/kg or subcutaneous flubendazole injections once with 2 or 10mg/kg or for five 5 days (10mg/kg). (DOCX) [file pntd.0006320.s004.docx]
